# An emerging class of new therapeutics targeting TGF, Activin, and BMP ligands in pulmonary arterial hypertension

**DOI:** 10.1002/dvdy.478

**Published:** 2022-04-27

**Authors:** Paul D. Upton, Benjamin J. Dunmore, Wei Li, Nicholas W. Morrell

**Affiliations:** ^1^ Department of Medicine University of Cambridge School of Clinical Medicine, Addenbrooke's and Royal Papworth Hospitals Cambridge UK

## Abstract

Pulmonary arterial hypertension (PAH) is an often fatal condition, the primary pathology of which involves loss of pulmonary vascular perfusion due to progressive aberrant vessel remodeling. The reduced capacity of the pulmonary circulation places increasing strain on the right ventricle of the heart, leading to death by heart failure. Currently, licensed therapies are primarily vasodilators, which have increased the median post‐diagnosis life expectancy from 2.8 to 7 years. Although this represents a substantial improvement, the search continues for transformative therapeutics that reverse established disease. The genetics of human PAH heavily implicates reduced endothelial bone morphogenetic protein (BMP) signaling as a causal role for the disease pathobiology. Recent approaches have focused on directly enhancing BMP signaling or removing the inhibitory influence of pathways that repress BMP signaling. In this critical commentary, we review the evidence underpinning the development of two approaches: BMP‐based agonists and inhibition of activin/GDF signaling. We also address the key considerations and questions that remain regarding these approaches.

## Key Findings

- Dysregulated bone morphogenetic protein (BMP) and activin signaling are associated with the pathophysiology of pulmonary arterial hypertension. Emerging therapies are aimed at these pathways. BMP9–based agonist therapies may represent a means to target the genetic and pathway defects in BMP signaling. Sotatercept targets upregulated activin signaling. Both approaches have the potential to generate disease–transforming therapies.

## 1 INTRODUCTION

Pulmonary arterial hypertension (PAH) is a rare disorder characterized by a progressive increase in the blood pressure in the arteries of the lungs, eventually causing death due to right heart failure. Prior to the development of therapies for treating PAH, the median survival was 2.8 years with a 5‐year survival rate of 34%.^1^ At the present time, as more therapies have been developed and licensed for treating PAH, the median survival currently stands at approximately 7 years overall.^2^, ^3^, ^4^ In the current classification system for pulmonary hypertension (PH), which encompasses all diseases characterized by elevated mean pulmonary arterial pressure (mPAP) at rest, PAH is classified as Group 1 PH.^4^ PAH is defined as precapillary PH in the absence of other causes, such as intrinsic left heart disease (Group 2 PH), lung disease (Group 3 PH), pulmonary artery obstructions (Group 4 PH), or unclear/multifactorial mechanisms (Group 5 PH).

PAH can be classified as idiopathic (IPAH) or heritable (HPAH) or can be associated with other factors such as drugs, toxins, connective tissue disease, HIV, liver disease, congenital heart disease or infection with schistosomiasis. Ultimately, these initiating events lead to similar pathologies. However, from a translational perspective, the underlying molecular mechanisms may differ, potentially warranting different therapeutic approaches in certain disease types.

### 1.1 Cellular pathology of PAH

Pathologically, PAH is characterized by a progressive thickening of the medial layers of the walls of distal muscular arteries (70‐550 μm diameter) and the muscularization of previously non‐muscular arterioles (<70 μm diameter).^5^ At the cellular level, multiple events may drive disease progression and are summarized in detail in recent reviews.^5^, ^6^ Medial expansion is thought to be primarily due to smooth muscle cell hyperplasia and hypertrophy.^5^ The endothelial layer lining the lumen experiences cellular loss due to damage and an increased susceptibility to apoptosis, certainly in the context of *BMPR2* mutations.^7^, ^8^ Endothelial damage and dysfunction can induce the release of factors that promote pulmonary artery smooth muscle cell expansion as well as inflammatory cell recruitment and infiltration.^9^, ^10^, ^11^ Flow sensing by endothelial cells in all vascular beds is important for the maintenance of endothelial polarity, barrier function, anti‐inflammatory responses, and homeostatic vasoregulator signalling. Contrasting with the systemic circulation (systemic vascular resistance = 900‐1200 dynes.sec.cm^−5^), the normal pulmonary circulation is a low pressure/low flow circuit (normal pulmonary vascular resistance = 100‐200 dynes.sec.cm^−5^). PAH patients exhibit reduced blood flow and wall shear stress that may exacerbate the disruption of normal vessel architecture.^12^, ^13^

Intimal lesions, representing eccentric intimal thickening and fibrotic, plexiform, concentric and dilation/angiomatoid lesions account for the reduced luminal area of small pulmonary arteries.^5^ In addition, there is obvious organisation of smooth muscle cells and myofibroblasts into concentric “onion skin” layers. Whether a single cell type contributes to intimal lesions, or multiple cell types are involved is still under debate, as is the relative impact of endothelial‐ or smooth‐muscle‐derived lesions.^5^ Recent evidence suggests that endothelial to mesenchymal transition may contribute, potentially driven by BMPR‐II deficiency.^14^, ^15^ A study in 2012 that employed adenoviral gene delivery to restore endothelial BMPR‐II signalling and improve hemodynamics in mice indirectly implicated EndMT inhibition.^16^ More recently, EndMT in PAH has been linked to increased endothelial HMGA1 expression arising from BMPR‐II loss of function.^17^ For a comprehensive insight into on EndMT in PAH, we refer the reader to a recent review by Gorelova et al.^18^ In addition to EndMT, differentiation of pericytes into smooth muscle cells^19^ and the potential for local and/or circulating progenitor cells to differentiate into endothelial or smooth muscle lineages^20^ add more dimensions to the abnormal changes in tissue architecture. In addition to abnormal changes in cell differentiation states and behaviour, the normal matrix structure and composition become dramatically altered. Ultimately, these changes lead to narrower and less compliant vessels. In addition to the structural changes, PAH is also associated with increased vasoconstriction at least in the earlier stages of disease, driven by reduced activity of the vasodilatory prostaglandin and nitric oxide pathways and an overall increased activity of the vasoconstrictive endothelin‐1 pathway.^21^

### 1.2 Current therapies for PAH

Several therapies have been developed to treat PAH, the majority of which target the vasoconstrictive component and are comprehensively summarized in Lau et al.^21^ A small subset of patients (6.8%)^22^ respond to calcium‐channel blockers and their disease can be managed long‐term on this therapy (Group 1.5.), though the mechanism underlying the responses in this subset is not known.^4^ The other three pathways targeted in PAH are the nitric oxide, prostacyclin, and endothelin‐1 pathways.^23^ The nitric oxide pathway is targeted using inhaled nitric oxide, sildenafil/tadalafil (to block cGMP degradation by phosphodiesterase‐5) or most recently, riociguat to activate soluble guanylate cyclase. In particular, sildenafil has been used with benefits, including improved haemodynamics.^24^, ^25^ Prostacyclin is an endogenous prostanoid synthesized by vascular endothelial cells that mediates vasodilation via prostanoid/IP receptors. However, the half‐life of prostacyclin is 42 seconds, so efforts have focused on developing analogues with longer half‐lives such as epoprostenol (*t*_1/2_ = 2‐3 minutes), iloprost (*t*_1/2_ = 20‐30 minutes), sodium beraprost (*t*_1/2_ = 35‐40 minutes), and treprostinil (*t*_1/2_ = 2‐3 hours). Although these drugs represent a substantial benefit to PAH patients, there are limitations to their effectiveness or risks due to the mode of delivery. Infections may develop in the indwelling catheters for continuous infusion of epoprostenol and there is a risk of catheter movement or pump failure. To overcome the difficulties associated with prostanoid metabolism, selexipag, a non‐prostanoid selective IP receptor agonist, was developed. However, the common side‐effects of headache and jaw pain are similar to those of the prostanoids. The endothelin pathway is targeted using the endothelin receptor antagonists, bosentan, macitentan, and ambrisentan. Patients can develop a gradual resistance to vasodilator therapies, leading to a requirement for dose escalation over time, thus increasing the risk of adverse effects. In some patients, vasodilators may stall disease progression but if resistance develops, the disease trajectory matches that of untreated patients. To overcome this, patients may be treated with combinations of 2 or even 3 drugs targeting different pathways and this regimen has proven highly beneficial to some patients.^23^, ^26^, ^27^ However, this approach is associated with an increased risk of side effects, requiring careful monitoring and prompt action should these arise.^27^ For example, the concurrent use of PDE‐5 inhibitors with riociguat is not recommended..^28^ Currently, it is difficult to foresee a transformational therapy arising from the vasodilator drug space. Histological assessment of lungs from patients who have been treated with vasodilators do not exhibit any substantial reduction of the pathological vascular remodeling,^29^ contrasting with data from preclinical studies targeting these pathways. The absence of a curative therapy for PAH indicates there is an unmet need for new therapeutic approaches that directly target the pathological remodeling underlying PAH. These alone, or in combination with current vasodilator therapies, could represent a new generation of transformative drugs in PAH.

## 2 PATHOGENIC MUTATIONS IN HUMAN PAH ARE SUGGESTIVE OF DISRUPTED BMP SIGNALING

### 2.1 Genetic mutations in PAH identify a role for loss of BMP signaling in PAH

In 2000, pathogenic mutations in *BMPR2*, the gene encoding the bone morphogenetic protein type‐II receptor (BMPR‐II) was identified as the major cause of PAH.^30^, ^31^ Experimental evidence from histological staining of lung tissues from PAH patients confirmed that BMPR‐II protein was reduced in PAH patients with or without *BMPR2* mutations.^32^ Furthermore, BMPR‐II protein and mRNA levels are reduced in rat models of PAH induced by hypoxia, monocrotaline (MCT), or Sugen(SU5416)‐hypoxia (SuHx)^33^, ^34^ and genetically modified mice carrying human *BMPR2* mutations develop PAH.^35^, ^36^

To date, 668 PAH‐associated mutations have been identified in the *BMPR2* gene,^37^ accounting for over 75% of heritable/familial PAH patients^37^, ^38^ and between 11%^39^ to 40%^40^ of IPAH patients. Further genetic studies with larger PAH cohorts have revealed more genes with potentially pathological mutations, each representing small but significant proportions of patients. These genes include *ACVRL1* (encoding ALK1), *ENG* (encoding endoglin), *GDF2* (encoding BMP9), *BMP10*, *SMAD9* (Smad8), *CAV1*, *KCNK3*, *AQP1*, *KDR*, *ATP13A3*, *TBX4*, and *SOX17*.^41^, ^42^, ^43^, ^44^, ^45^, ^46^, ^47^, ^48^, ^49^, ^50^, ^51^ Of note, the expression of many of these genes is enriched in, or specific to vascular endothelial cells. Intriguingly, mutations in *ACVRL1* and *ENG* are more commonly associated with hereditary hemorrhagic telangiectasia (HHT) than PAH. Moreover, the presentation of these diseases can overlap in some patients, as summarized in Hodgson et al.^52^ In both PAH and HHT, the endothelial cell is considered the pivotal cell type directly impacted functionally by these mutations. Changes in SMC function may be impacted as a secondary consequence in PAH and HHT, though in the context of BMPR‐II mutations in PAH, reduced BMPR‐II in smooth muscle cells also promotes functional defects.^53^ As with all genetic studies, the confirmation of pathogenicity of variants in the genes listed above requires supporting experimental evidence via a combination of approaches including, but not restricted to, further human studies and functional studies in animal models and cell‐based systems.

### 2.2 Components and regulation of BMP and TGFβ signaling complexes

**FIGURE 1:**
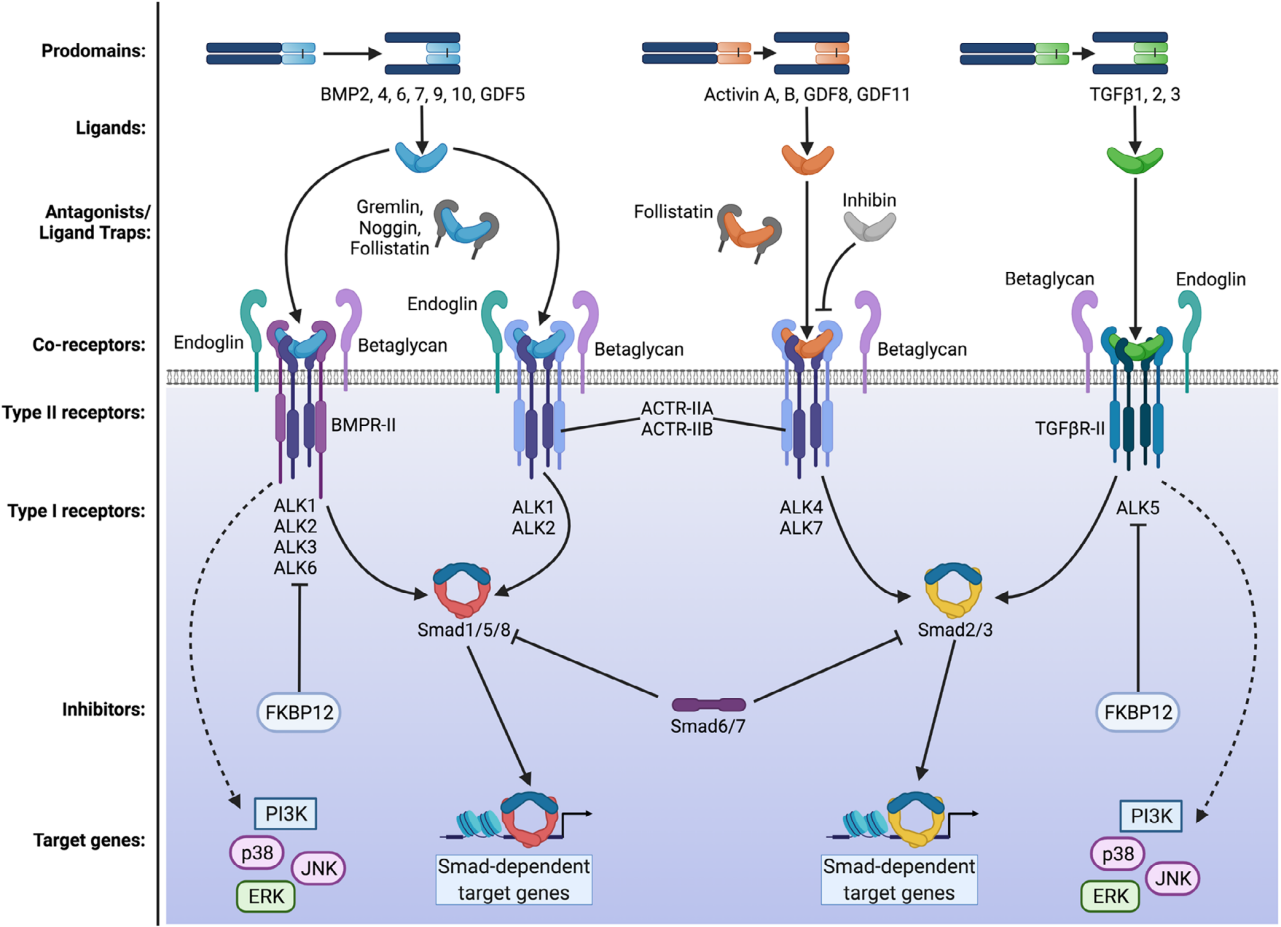
Schematic depicting the multiple levels of regulation of bone morphogenetic protein (BMP), growth and differentiation factor (GDF), activin and transforming growth factor‐β (TGFβ) signaling pathways. The activity of the ligands is regulated via secretion and processing. The prodomains are cleaved from the active growth factor domain (GFD) by furin‐type prohormone convertases. In the case of BMP9 and BMP10, the non‐covalent complex of the prodomain and GFD are biologically active. The availability of the GFDs for receptor binding is also regulated by local concentrations of soluble inhibitory ligand traps (eg, Gremlin, Noggin, Follistatin). Ligand selectivity is regulated and refined by the cell surface composition of complexes comprising Type‐I receptors (ALK1‐7), Type‐II receptors (BMPR‐II, ACTR‐IIA, ACTR‐IIB, TGFβR‐II) and co‐receptors (endoglin, betaglycan). Each receptor complex has selectivity for specific ligands ranging from high affinity, through lower affinities to no detectable interaction. The cytoplasmic serine/threonine kinase domains of the activated receptor complexes phosphorylate and activate canonical Smad complexes and non‐canonical kinases. Smad1/5/8 typically mediate BMP/GDF5 signaling and Smad2/3 typically mediate activin/GDF8/GDF11/TGFβ signaling, though some ligands can activate both pathways as referred to in Section 2.2. For simplicity, activation of multiple pathways (e.g., TGFβ_1_ signaling via ALK1 and ALK5) are not shown in this figure. Smad6 and Smad7 act as inhibitory Smads. Active Smad complexes translocate to the nucleus and alter the transcription of target genes to regulate cell functions. Created with BioRender.com

Bone morphogenetic proteins (BMPs) are members of the TGFβ superfamily of cytokines. Over 33 genes encode TGFβ family ligands, which can be broadly divided into three subfamilies, the TGFβs, the activins, and the BMPs/Growth differentiation factors (GDFs). The ligands are generally synthesized as a proprotein chain comprising the prodomain and growth factor domain (GFD). During processing, the GFD dimerizes and the prodomains are generally cleaved from the GFDs by furin‐type prohormone convertases but remain associated with the GFD. The GFD homodimers represent the active receptor binding subunit, and these ligands initiate signaling by forming hexameric complexes with cell surface receptors comprising two type‐I receptors and two type‐II receptors, all of which are serine threonine kinases. Upon signaling complex formation, the constitutively active type‐II receptors phosphorylate the type‐I receptor at the GS‐domain. This activates the type‐I receptor, which then phosphorylates signaling mediators such as the canonical receptor‐regulated SMADs (R‐SMADs) and non‐canonical intracellular kinases (Figure 1). The most striking aspect of TGFβ family signaling complexes is that although the ligands are numerous, the available cell surface receptors comprise only 7 type‐I receptors and 5 type‐II receptors and canonical signaling hones down to only two families of R‐SMADs, of which Smad1/5/8 mediates mostly the BMP signals, and Smad2/3 mediates the signals from TGFβs and activin family ligands (Figure 1). Although the overall scheme shown in Figure 1 delineates the Smad signaling pathways to provide clarity, there are circumstances whereby both signaling pathways might be activated. For example, BMP9 stimulates C‐terminal phosphorylation of Smad1/5/8 with high activity (0.01 ng/mL) and Smad2 with low affinity (1 ng/mL) in endothelial cells.^54^ Conversely, TGFβ_1_ can mediate activation of both Smad2/3 and Smad1/5/8 in endothelial cells, with ALK1‐dependent Smad1/5/8 signaling being dependent on ALK5 whilst inhibiting ALK5‐dependent Smad2/3 activity.^55^

BMP signaling complexes are regulated at multiple levels (Figure 1). First, the ligands are secreted as the GFDs in complex with their prodomains, which cover the receptor binding sites on the GFDs, thus requiring the prodomains to be displaced or dissociated for receptor binding and signaling. Within the TGFβ superfamily, the prodomains differ in the mechanisms by which they regulate the availability of their cognate GFDs. For example, the TGFβ_1_ prodomain strongly associates with the GFD and in this small latent complex form, it prevents the GFD binding to TGF receptors. TGFβ_1_ activation requires covalent interactions with the extracellular matrix and cellular contractile forces to release the ligand.^56^ GDF8 and GDF11 are also rendered latent via association with their prodomains and are activated by a second cleavage event mediated by BMP‐1/Tolloid.^57^, ^58^ Though BMP9, BMP10, and Activin A are also secreted as non‐covalent complexes with their prodomains, these complexes are not latent and signal with similar potencies to the free GFDs.^59^, ^60^, ^61^ Second, there is a high degree of promiscuity in the receptor: ligand interaction. Many BMPs can signal through multiple receptor pairs and many receptors can form different receptor pairs to mediate signaling, even from the same ligands (Figure 1). As each ligand has different affinities for particular type‐I and type‐II receptors, the concentration of ligand is important. At lower concentrations, a BMP ligand will only bind its cognate receptor complexes with high affinity to induce signaling. As the concentration of ligand increases, receptor complexes with lower affinity may also be activated. Furthermore, competition for the same receptor by ligands of differing affinities could influence the signaling outcome. Third, several co‐receptors, such as endoglin and RGMs, bind ligands at the receptor binding site and modify downstream signaling. Last, there are many circulating ligand traps can bind ligands tightly at the receptor binding surface, preventing the signaling complex formation, such as Noggin and follistatin. Therefore, at any one time, the overall signaling result is determined by the presence and relative quantities of ligands, the cell surface receptors, co‐receptors, and the ligand traps.

### 2.3 Targeting BMP signaling for treating PAH

As *BMPR2* mutations are the most established and prevalent genetic cause of PAH, many different approaches have been explored to target BMP signaling in treating PAH, mostly by restoring cell surface BMPR‐II expression and/or enhancing BMP signaling. These include Tacrolimus/FK506 which has been explored in preclinical models and in clinical trials. Its mode of action is to enhance BMP signaling by removing the intracellular suppressor FKBP12 from binding to the type‐I receptors (Figure 1). Hydroxychloroquine and 4‐phenylbutrrate (4PBA) are under investigation for enhancing cell surface BMPR‐II expression, and Etanercept has shown efficacy in preclinical models of PAH by preventing the loss of cell surface BMPR‐II on PASMCs. Details of these approaches have been summarized in our recent review.^62^ Two additional approaches which target the ligands in the signaling complex have shown promising results in preclinical models and/or clinical trials, namely: (a) the BMP9 agonist approach which specifically enhances endothelial BMP signaling, and (b) sotatercept, a ligand trap for activin family ligands. Both approaches are under intense drug development programmes as first‐in‐class therapies for PAH. In the remainder of this commentary, we will provide critical reviews and commentaries on these two approaches.

## 3 PATHWAY‐DIRECTED THERAPEUTIC APPROACHES

### 3.1 Targeting BMP Signaling for treating PAH

Since the establishment of the critical role of BMPR‐II loss of function in the pathogenesis of PAH, significant efforts have focused on understanding how BMPR‐II mutations and dysfunction cause PAH and how to target such a pathway to treat PAH. BMPR‐II is most highly expressed in the pulmonary endothelium, though pulmonary artery smooth muscle cells also express BMPR‐II.^32^ Early studies in PASMCs revealed reduced BMP2, BMP4, BMP6, and BMP7 signaling as a consequence of *BMPR2* haploinsufficiency.^63^, ^64^ However, genetic evidence in mice suggests that BMP2 signaling contributes more to normal vascular signaling and function,^65^ whereas BMP4 may be biased toward regulating airway function.^66^ Also, substantial reduction of BMPR‐II in PASMCs by full knockout, siRNA, or haploinsufficiency combined with BMPR‐II repression by TNFα change the dynamics of BMP6/7 signaling, leading to pathogenic signaling via ALK2/ACTR‐IIA.^34^, ^64^, ^67^

**FIGURE 2:**
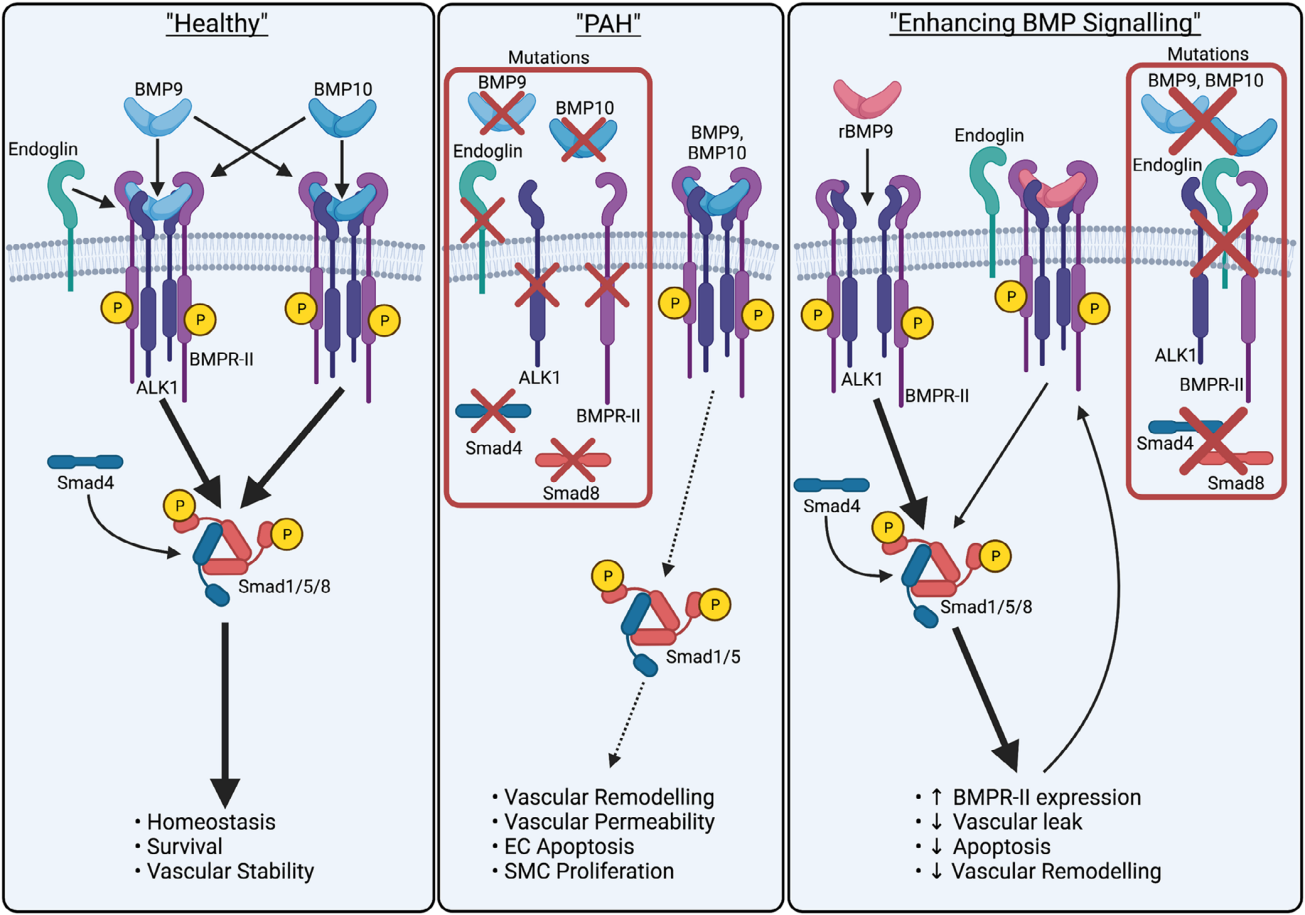
Rationale of BMP9‐based therapies in PAH. *Left panel*: Circulating BMP9 and BMP10 signal via high‐affinity endothelial cell surface receptor complexes comprising ALK1 and BMPR‐II with the co‐receptor, endoglin. This continuous signaling mediates normal Smad1/5/8 activity to maintain endothelial cell homeostasis. *Centre panel*: Genetic reduction of functional ALK1, BMP9, BMP10, BMPR‐II, endoglin, Smad4 or Smad8, exacerbated by one or more “second hits,” critically reduces endothelial BMP9/BMP10 signaling and results in the pathological changes underlying the development of PAH. *Right panel*: Proposed mode of action of supplementation with exogenous recombinant BMP9 (rBMP9), leading to restored endothelial cell signaling via enhancement of BMPR‐II protein levels and normalisation of endothelial cell functions. Created with BioRender.com

The genetic architecture of PAH (Figure 2) strongly suggests that endothelial cells represent the pivotal cell type in which loss of **BMPR‐II** drives PAH (key proteins mutated in PAH in bold). Of the other receptors in the TGF superfamily, rare mutations in the genes encoding the type‐I receptor *ACVRL1* (**ALK1**) and the accessory receptor *ENG* (**endoglin**) have been identified. Like BMPR‐II, both of these proteins are highly expressed in the pulmonary endothelium^68^ and ALK1 expression in adult rat lung is 10‐200‐fold higher than its expression in other tissues, suggesting ALK1 preferentially functions in pulmonary blood vessels. ALK1 and BMPR‐II constitute the endothelial receptors for the circulating ligands, **BMP9** and **BMP10** (Figure 2), with an EC_50_ of 10‐50 pg/mL in both arterial and microvascular endothelial cells.^54^, ^69^, ^70^, ^71^ Endoglin represents an endothelial BMP9/10 co‐receptor. Of note, we have shown that ACTR‐IIA can also mediate endothelial BMP9 signaling.^54^ There is redundancy in some functions of these type‐II receptors, such that Smad1/5 phosphorylation and transcriptional induction of *ID1* and *ID2* are attenuated only if expression of both BMPR‐II and ACTR‐IIA is reduced.^54^ However, other transcriptional targets, such as *IL8* and *SELE*, are solely dependent on BMPR‐II.^54^ The activated ALK1/BMPR‐II complex mediates canonical signaling via phosphorylation and activation of Smad1/5 proteins,^54^, ^69^, ^70^, ^72^ which then associate with Smad4 to form a transcriptional complex that targets BMP responsive genes, such as *ID1* and *ID2*.^73^ **Smad9** may contribute to canonical signaling, but may also function as an inhibitory Smad.^74^ In addition to direct BMP signaling, the protein products of many other PAH‐related genes either interact with the receptor or signaling complex directly (such as CAV1), or are regulated by BMP9 or BMP10 signaling in endothelial cells (such as AQP1 and KDR).

BMP9 and BMP10 are reported to promote vascular quiescence in endothelial cells at concentrations measured in plasma.^75^, ^76^ Indeed, BMP9 signaling via BMPR‐II is considered to promote endothelial cell homeostasis and quiescence.^75^ Loss of BMPR‐II predisposes human pulmonary artery endothelial cells (hPAECs) to apoptosis^7^ and BMP9 can prevent hPAEC apoptosis induced by serum starvation.^36^ This may be related to impaired mitochondrial function and increased DNA damage^77^ along with a failure to undergo normal DNA repair.^78^ BMPR‐II loss also leads to increased permeability of the hPAEC monolayer.^36^, ^79^ Recently, we reported that depletion of BMP9 alone from culture medium containing FBS increases PMEC monolayer permeability and promotes apoptosis.^71^ In vivo, loss of BMPR‐II function reduced endothelial barrier function in vivo and the development of PAH in preclinical models.^36^ Subacute administration of neutralizing BMP9 antibody in healthy adult mice results in vascular leak, pulmonary vascular oedema, and inflammation.^71^ These data are potentially important in the context of reduced vascular perfusion and wall shear stress in PAH.^12^, ^13^ The maintenance of endothelial Smad1/5 signalling in vivo is dependent on BMP9 (and probably BMP10 signalling) via ALK1^75^, ^76^ and BMP9‐ALK1 mediates endothelial flow sensing.^80^ Reduced perfusion may limit the availability of BMP9/BMP10 and contribute to the reduction of pulmonary vascular Smad1/5 phosphorylation observed in PAH patients,^63^ leading to a loss of normal endothelial homeostasis.

Further support for loss of endothelial BMP9/10 signaling is provided by the identification of pathogenic mutations in the *GDF2* (BMP9) and, to a lesser extent, *BMP10* genes in some PAH patients. Intriguingly, PAH patients with *GDF2* mutations exhibit reduced plasma levels of both BMP9 and BMP10 and reduced plasma‐derived BMP9/10 endothelial activity.^46^, ^47^, ^49^, ^50^ The reduction of both BMP9 and BMP10 in plasma appears to be due to a strong correlation between the levels of these ligands, although the levels are not always tethered as some individuals with low BMP9 have normal BMP10 levels and vice versa.^81^ We recently reported a child who was homozygous for a truncating *GDF2* mutation who exhibited undetectable plasma BMP9 and BMP10 and an absence of serum BMP9/BMP10 activity.^82^

### 3.2 Agonist‐based BMP therapy

Ultimately, enhancing BMPR‐II levels and BMP signaling leading to restoration of normal endothelial function is the aim of agonist therapies (Figure 2). On the basis that BMP9 induces *BMPR2* expression in endothelial cells,^54^, ^69^ we tested the hypothesis that administration of exogenous BMP9 would be beneficial in PAH.^36^ Indeed, in mouse models of *Bmpr2* deficiency or rat PAH models, BMP9 administration reduced the severity of PAH with a dramatic reduction of vascular remodeling.^36^ In cellular models, BMP9 restored monolayer integrity (represented as reduced permeability), reduced apoptosis, and restored endothelial quiescence.^36^ Although BMP9 primarily signals with high affinity via ALK1 and BMPR‐II, there is a risk of signaling via low‐affinity type‐I receptors such as ALK2, and the alternative type‐II receptors, ACTR‐IIA and ACTR‐IIB.^83^ For example, BMP9 signaling via low‐affinity receptors (>1 ng/mL) promotes TNFα‐dependent monocyte recruitment to aortic endothelial cell monolayers and neutrophil recruitment to LPS‐treated monolayer of pulmonary artery endothelial cells.^84^ However, it is notable that the measured levels of BMP9 in plasma are generally lower than 1 ng/mL. Furthermore, if systemic LPS levels are elevated, then BMP9 production by the liver is decreased,^71^, ^85^ so the role of low‐affinity receptors mediating BMP9‐dependent inflammation may be reduced. As aberrant inflammation is a hallmark of PAH,^86^ the interaction with BMP9 is an important consideration. Of note, both the SuHx and MCT rat models of PAH exhibit strong hallmarks of inflammation,^87^, ^88^ yet exogenous BMP9 administration proved beneficial in these models and reduced inflammation.^36^

### 3.3 The paradox of BMP9 inhibition in animal models of pulmonary hypertension

**FIGURE 3:**
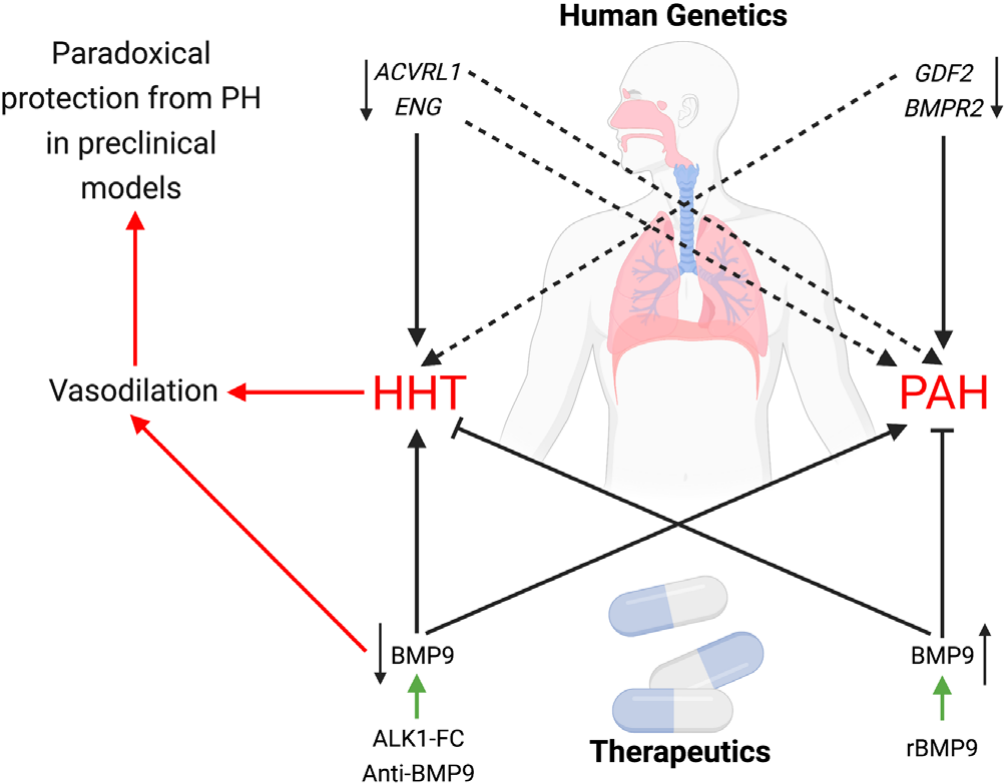
Overlapping genetic architecture of mutations underlying PAH and HHT may explain the paradoxical observations of alleviation of PAH in preclinical models by BMP9 agonist therapy and BMP9 blockade. *BMPR2* mutations underly PAH in man, whereas *GDF2* (BMP9) mutations are associated with PAH or syndromes with an HHT‐like phenotype. Mutations in *ACVRL1* (ALK1) and ENG most commonly cause HHT and less frequently cause PAH. In preclinical models, recombinant BMP9 (rBMP9) agonist therapy dramatically reduces the PAH phenotype and is predicted to exert beneficial effects in preclinical HHT models. Inhibition of BMP9 with anti‐BMP9 or inhibiting BMP9 and BMP10 with ALK1‐Fc in preclinical rodent models of PAH may cause vasodilation and lead to a paradoxical protection from PAH due to the induction of an HHT‐like phenotype. Created with BioRender.com

Recently, two studies have been published by the same group suggesting that BMP9 partially contributes to hypoxia‐induced pulmonary hypertension.^89^, ^90^ These studies have primarily focused on studies of *Bmp9* ^−/−^ mice in normoxia and hypoxia, supported by studies of administration of a BMP9 neutralizing antibody in wild‐type mice.^89^ There still remain some key questions regarding differences between the *Bmp9* ^−/−^ mice and human PAH. Developmentally, *Bmp9* ^−/−^ mice display reduced basal muscularization of their pulmonary arteries,^89^, ^90^ so the blunted muscularization response to hypoxia compared to wild‐type animals may reflect different pulmonary vessel structures. Estimation of the fold‐change in remodeling relative to baseline in animals of the same genotype suggests that the extent of remodeling is similar between wild‐type animals and *Bmp9* ^−/−^ mice. Also, administration of a neutralizing BMP9 antibody might be confounded by the biology of the system in this context. If BMP9:BMP10 heterodimers are a major circulating form, the administration of a BMP9 neutralizing antibody may inhibit signaling by both BMP9 and BMP10, potentially eliciting a more dramatic impact than would be anticipated from solely inhibiting BMP9.^91^ Indeed, IP of human plasma with this antibody leads to a partial reduction of BMP10 levels detected by ELISA, either confirming the presence of heterodimers or association of BMP9 and BMP10 with a common carrier protein.^81^ Also administration of the ligand trap ALK1‐ECD, which inhibits both BMP9 and BMP10, blunted the pulmonary hypertension in the rat MCT and SuHx models.^89^ Indeed, this may produce a mixed phenotype reflecting the high‐output heart failure observed in *Bmp9* and *Bmp10* double‐knockout mice.^90^ This observation is contrary to a reported worsening of hypoxic pulmonary hypertension in rats injected with ALK1‐ECD.^92^ There may be further confounding effects of BMP9 inhibition in preclinical models, with evidence provided from human disease. Administration of Dalantercept, a fusion protein comprising the soluble extracellular domain of human ALK1 fused to a human IgG1 Fc domain, to oncology patients causes the emergence of telangiectasias as an adverse effect.^93^ This suggests that blockade of BMP9 and BMP10 may predispose to an HHT phenotype in preclinical models (Figure 3). Also, if BMP9 and BMP10 promote the maintenance of normal vascular tone via induction of ET‐1 and inhibition of adrenomedullin and apelin, BMP9 and BMP10 blockade may increase vasodilatation, especially when considering the substantial inhibition of adrenomedullin expression in endothelial cells.^89^ Ultimately, there is a disconnect between the reported role of endogenous BMP9 in rodent disease phenotypes and the genetic data from patients, as both heterozygous and homozygous BMP9 mutations are associated with PAH.^46^, ^47^, ^49^, ^50^ Currently, we would argue that administration of exogenous BMP9 is beneficial in rodent models of PAH and has the potential to translate to a biologics‐based therapy in human PAH.^36^

### 3.4 Factors to consider in BMP9 and BMP10 administration

There may be a risk that exogenous BMP9 or BMP10 administration may cause other side effects such as increased local or systemic calcification.^94^ The risk of a local injection site event, such as ectopic calcification should be considered, especially if repeated administrations of BMP9/10 are needed. In our previous study with BMP9, gross examination of the injection site following chronic daily administration did not reveal evidence for calcification.^36^ Aberrant BMP2, BMP4, and BMP6 signaling are most strongly associated with plaque instability and vascular calcification in atherosclerosis.^95^, ^96^, ^97^ A study by Kang et al. of the contribution of adenovirally‐delivered BMPs to heterotopic bone formation in mice indicated that BMP9 exerts robust bone‐forming activity when compared to other BMPs.^98^ Of note, the context of this response to BMP9 may be more important than for other BMP ligands, as muscle damage is required for BMP9‐induced ossification, whereas BMP2 induces a response irrespective of the presence or absence of damage.^99^ The data published by Kang et al. showed an absence of heterotopic ossification to the BMP10 adenovirus.^98^ However, the limitation of this study was that processing of the proproteins to the active BMP ligands was not confirmed and the BMP10 expression may not have translated to mature protein production. Indeed, recent studies of direct injection of wild‐type BMP9 or BMP10 recombinant protein into skeletal muscle have shown that both induce heterotopic ossification.^100^ Therefore, this possible side‐effect cannot be excluded and the generation of variants lacking osteogenic activity is a key consideration.

### 3.5 Reducing activin family signaling with ligand traps

**FIGURE 4:**
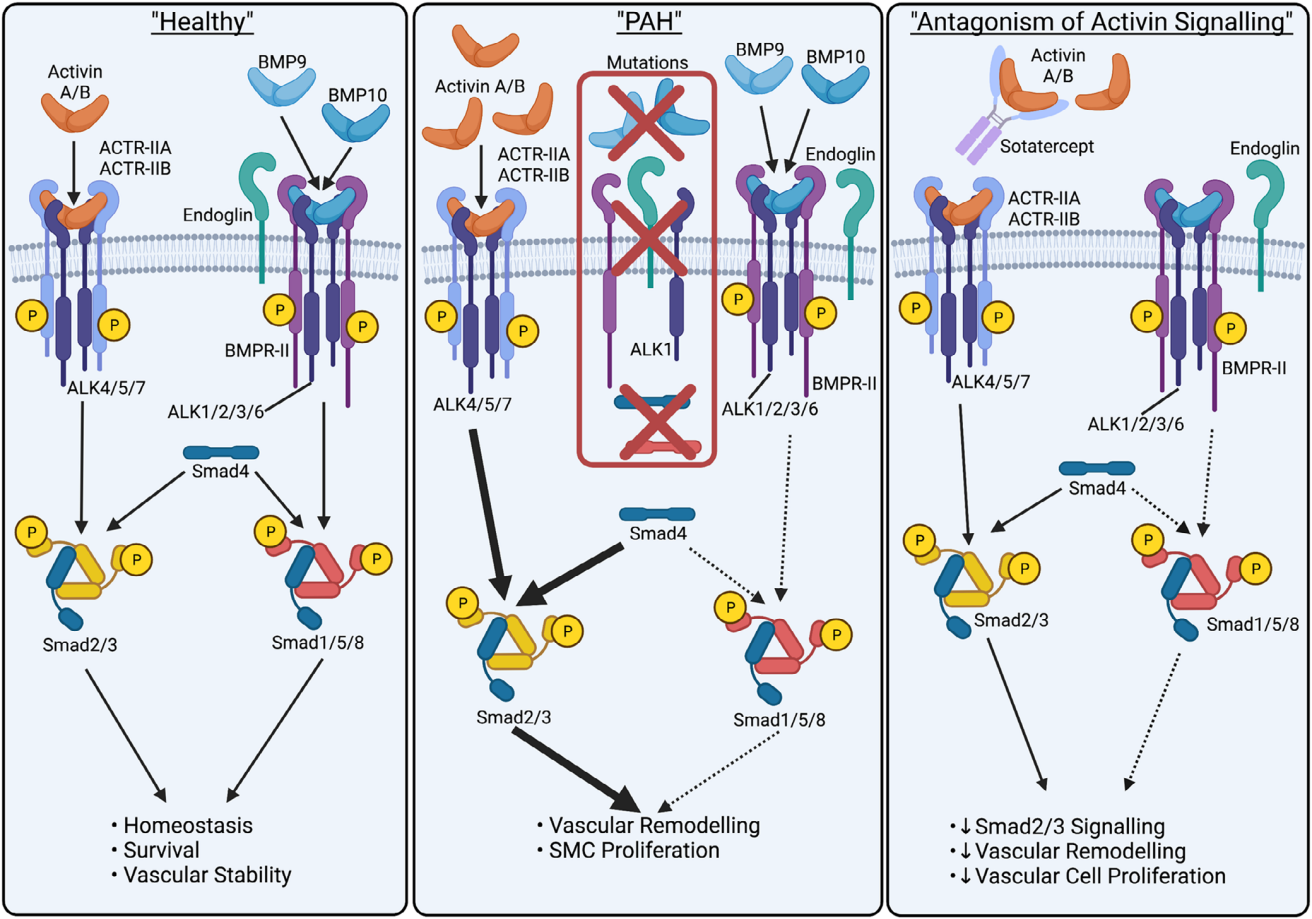
Possible rationale for sotatercept therapy in PAH. *Left panel*: Signaling by activin and BMP9/BMP10 via their respective endothelial cell surface receptor complexes maintains endothelial cell homeostasis. *Centre panel*: Genetic reduction of functional ALK1, BMP9, BMP10, BMPR‐II, endoglin, Smad4, or Smad8 lead to reduced BMP signaling. The reduction of BMP signaling may be exacerbated by one or more “second hits” that may also cause an elevation of activin A production. The excessive activin A signals and Smad2/3 signaling may promote the pathological changes underlying the development of PAH. *Right panel*: Sotatercept acts as a ligand trap to neutralize activin and reduce aberrant Smad2/3 signaling. Created with BioRender.com

PASMCs from PAH patients show a heightened response to TGFβ_1_ and ALK5 inhibition exerted a beneficial effect in the MCT rat PAH model.^33^ Similarly, TGFβ‐RII‐Fc, which acts as a ligand trap for TGFβ_1_ and TGFβ_3_, attenuated PAH in the rat models induced by either MCT or SuHx.^101^ Other ligands that induce Smad2/3 phosphorylation are activin family ligands, including GDF8, GDF11, Activin A and Activin B, which all bind to ACTR‐IIA and ACTR‐IIB with high affinity (Figure 4). Interestingly, ACTR‐IIA‐Fc demonstrated efficacy in reversing PAH in both MCT and SuHx‐induced rat models of PAH.^102^ A human ACTR‐IIA‐Fc‐based therapy, sotatercept, has progressed furthest in the clinic and could represent a significant advance in this new class of therapies for PAH. Results from the recent Phase‐II PULSAR trial reported a reduction of PVR in both the lower (0.3 mg/kg) and higher (0.7 mg/kg) doses.^103^ Based on the PULSAR data, the United States Food and Drug Administration (FDA) has granted designations of Orphan Drug and Breakthrough Therapy status to sotatercept for the treatment of PAH. In addition, the European Medicines Agency (EMA) has granted Priority Medicines (PRIME) designation to sotatercept for the treatment of PAH. Currently, sotatercept is under investigation as a treatment for PAH in 3 Phase‐III trials.

### 3.6 Potential side effects of sotatercept

The initial success of sotatercept is encouraging, though like any emerging therapy, there is much to understand about the mode of action of this drug. This is of particular relevance to the adverse effects reported in the PULSAR trial.^103^ Although the trial reported serious adverse effects in 6% (2/32 patients) of patients receiving the 0.3 mg/kg dose, the incidence of serious adverse events increased to 24% (10/42 patients) in those patients receiving the 0.7 mg/kg dose.^103^ This raises the question of whether the increase in adverse events may be a consequence of the selectivity profile of sotatercept. A study of the affinities of the type‐II receptors for different ligands demonstrated that ACTR‐IIA‐Fc can bind to several ligands, in the order (Kd) Activin A (4.3e^−11^M) > Activin B (6.1e^−11^ M) > BMP10 (3.8e^−10^M) > GDF11 (5.7e^−10^M) > BMP7 (1.6e^−9^M) > BMP4 (3.5e^−9^M) > GDF8 (3.7e^−9^M) > BMP6 (1.1e^−8^M).^104^ If sotatercept has a similar binding profile, as the dose is increased, it is likely that sotatercept inhibits a broader range of ligands. Of particular concern is the potential for sotatercept to inhibit BMP10 signaling because BMP10 may be protective to the endothelium and ensure normal cardiac function by preventing cardiac fibrosis, similar to BMP9.^105^, ^106^ There also remains a question relating to the impact on other BMP ligands, such as BMP6 and BMP7, which may be involved in the maintenance of cardiac differentiation.^107^ Also, BMP6 regulates liver iron homeostasis and reduced BMP6 levels resulting from germline mutations underlie some cases of haemochromatosis.^108^ Indeed, the evidence for abnormal iron handling in PAH has accumulated over the past decade and iron deficiency is associated with a reduced 6‐minute walk distance.^109^ In the recently reported Phase‐II study with sotatercept, adverse effects included elevated serum haemoglobin and thrombocytopaenia, with higher frequency in those patients receiving 0.7 mg/kg.^103^ The elevation of haemoglobin by sotatercept is known and was proposed to be via rescue of the inhibition of late‐stage erythropoiesis by GDF11 and activin A.^110^ The contribution of GDF11 to erythropoiesis is not clear as *Gdf11* knockout mice were not protected against β‐thalassaemia or myelodysplastic syndromes.^111^, ^112^ Also, Activin A is reported to promote erythropoiesis in human bone marrow,^113^ but inhibition of activin A, GDF8, and GDF11 with a follistatin molecule increased muscle mass without altering blood cell count.^114^ This highlights a question regarding the contributions of these and other ligands in the regulation of erythropoiesis. Given the potential impact of sotatercept on iron handling, other markers such as plasma hepcidin and serum ferritin levels may warrant investigation. This may be of importance when considering patient cohorts, as plasma hepcidin levels are elevated in a subset of IPAH patients.^115^ Whether this is due to elevated signaling by BMP6,^116^ activin B,^117^ or other factors that can regulate hepcidin is uncertain. As there is heterogeneity regarding the hepcidin and iron status in PAH patients, the influence of sotatercept on circulating markers of iron homeostasis may provide insights into the efficacy and safety profiles in these patient subgroups with respect to iron overload and/or anaemia.

### 3.7 The mechanism of action of sotatercept is still unclear

**FIGURE 5:**
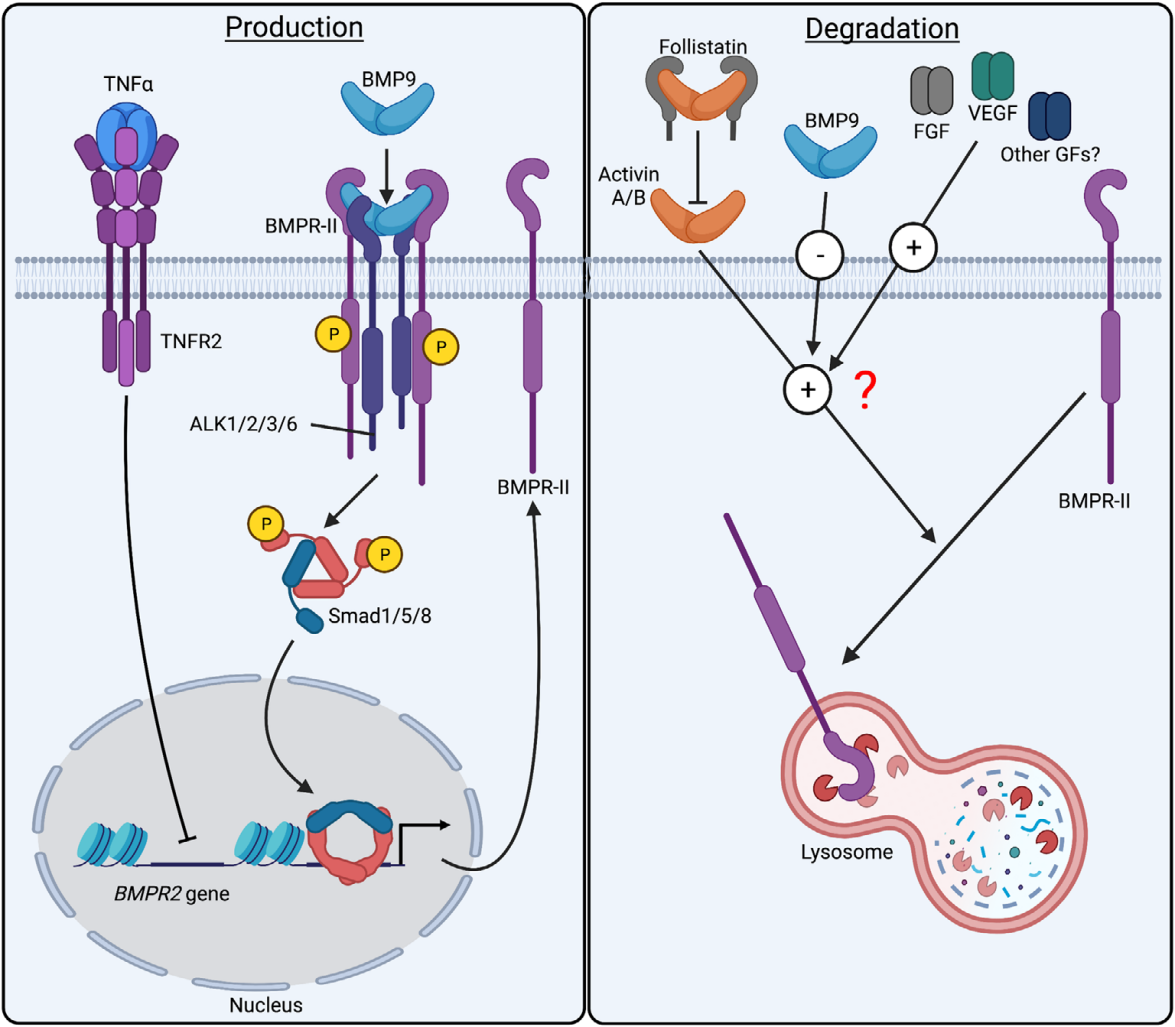
Multiple signalling inputs influence the net levels of cell surface BMPR‐II protein in endothelial cells. BMP9 promotes BMPR‐II transcription and increases cell surface BMPR‐II, whereas TNFα represses BMPR‐II transcription. Activin A may promote BMPR‐II protein degradation with possible enhancement of this effect by growth factors and inhibition by BMP9. Although circulating activin A levels are elevated in PAH patients, levels of the activin ligand trap, follistatin, are also elevated and, therefore, it is not known if plasma activin activity is increased. Created with BioRender.com

As sotatercept has the potential to target multiple ligands, it is still not certain which of these are most relevant to PAH pathobiology and to what extent the mechanism of action of sotatercept involves changes in BMP signaling. The proposed hypothesis for the mechanism of action is that activin disrupts BMPR‐II signaling and by blocking activin with sotatercept, this restores normal BMP signaling (Figure 4).^102^ This is suggested from a study showing that activin overexpression via retroviral transduction in pulmonary artery endothelial cells reduces BMPR‐II protein by accelerating the rate of degradation.^118^ However, in the same study, the reduction of BMPR‐II protein expression by exogenous activin was less dramatic, so whether retroviral overexpression is adversely affecting BMPR‐II turnover needs to be considered.^118^ Also, these studies of BMPR‐II protein reduction by exogenous activin seem to have been undertaken in growth media, which is supplemented with growth factors such as VEGF and FGF. Whether these growth factor pathways contribute to the observed degradation of BMPR‐II in cells exposed to activin is important to consider. In a separate study, exogenous activin A did not promote a reduction of BMPR‐II protein expression in endothelial cells exposed to BMP9,^119^ which induces endothelial BMPR‐II expression in both arterial and microvascular endothelial cells.^36^, ^54^, ^119^ Therefore, the levels of BMPR‐II expression, protein turnover, and downstream signaling in endothelial cells may be proportionate to the net effect of signals from BMP9, growth factors, and activin plus other pathways, such as TNFα (Figure 5).^34^

Plasma BMP9 levels are normal in the majority of PAH patients^81^ and although circulating activin A is elevated in PAH patients, levels of the endogenous activin antagonist, follistatin, are also increased.^120^ Therefore, it is not certain whether plasma activin A activity is increased, or if it is neutralized by the elevation of follistatin. However, local activin expression is increased in the lungs of PAH patients^102^ and BMPR‐II expression is reduced,^32^ so it is conceivable that the balance may be shifted in favour of activin signaling relative to BMP signaling.^120^ From preclinical studies, ACTR‐IIA‐Fc undoubtedly provided a beneficial effect on haemodynamics,^102^ although claims of restoration of BMP signaling are not supported by the preclinical data, which did not demonstrate an improvement of Smad1/5/8 signaling by ACTR‐IIA‐Fc.^102^ The currently available data provide strong support for sotatercept inhibiting pathogenic activin/GDF signaling, but not for restoration of BMPR‐II protein or downstream BMP signaling.

Whether the targeting of activin is the sole mechanism of action of sotatercept is also not definite. In addition to activin, increased expression of GDF8 (myostatin) and GDF11, the latter less pronounced, was demonstrated in PAH patient lungs.^102^ GDF11 expression is also increased in lung endothelial cells of hypoxic and SuHx rat models of PAH.^121^ Furthermore, endothelial cell‐specific *Gdf11* knockout protects mice from developing PAH in response to hypoxia or SuHx.^121^ Increased endothelial cell GDF11 expression was associated with enhanced endothelial cell proliferation and a reduction of the capacity to migrate and form tubes.^121^ However, a separate study suggested that GDF11 reduces endothelial apoptosis and inflammatory cell recruitment to stabilize atherosclerotic plaques in ApoE‐deficient mice,^122^ so the relative risk vs benefit of GDF11 inhibition may need to be considered in patients at risk of adverse cardiovascular events. Currently, the role of GDF11 in the biology of aging, particularly with relevance to muscle wasting, cardioprotection and the regulation of bone density is still unclear.^123^ Different studies with very similar designs have reported different outcomes and the results of some early studies have been questioned following the identification of non‐specificity of some reagents.^123^ A recent model proposed by Egerman et al. suggests that GDF11 levels are elevated in embryonic development and decline in adults as a normal homeostatic mechanism, with elevation of GDF11 in adults being associated with disease.^123^ Although the contribution of elevated GDF8/myostatin to the pathogenesis of PAH is less clear, blockade of GDF8/myostatin may provide benefit to patients to counter the reduced skeletal muscle mass and weakness reported in IPAH patients.^124^ However, blockade of ACTR‐IIB, a receptor for several ligands, including activin A, GDF8/myostatin and GDF11 reduced the metabolic capacity of skeletal muscle, reducing exercise tolerance.^125^ Thus, inhibition of GFD11 and GDF8 with sotatercept might contribute to the beneficial effects in PAH, but the pleiotropic roles of these ligands and Activin A in normal homeostasis warrant careful consideration and thorough investigation of the impact of their blockade.

There is one aspect of endogenous GDF8 and GDF11 regulation that is unclear regarding the possible inhibition of these ligands by sotatercept. Both GDF8 and GDF11 are secreted as latent proteins due to the presence of their prodomains,^57^, ^58^ and they require activation by a second cleavage by BMP‐1/tolloid metalloproteinases, possibly presented by target cells.^58^, ^126^ In cell studies, ACTR‐IIA‐Fc blocked Smad2 and Smad3 signaling in response to the GDF8 and GDF11 growth factor domains, in addition to inhibiting the activin A response.^102^ However, it is not known if sotatercept can bind to latent GDF8 or GDF11, or if it will inhibit these ligands when they are cleaved and activated at the cell surface. In the context of the vessel, it is also not known if endothelial cells secrete GDF11 at the apical or basolateral surface. If the latter is the case, GDF11 might be activated on the smooth muscle cell surface, which may not be accessible by sotatercept in the circulation. Therefore, exploration of the model of GDF11 and GDF8 secretion and activation in PAH could be vital to our understanding of its mechanism of action.

## 4 CONCLUDING REMARKS

In conclusion, therapeutic targeting of the TGF and BMP pathways represents the potential for a new generation of disease‐modifying drugs. As research continues into the intricacies of the mode of action of these drugs, we gain a broader experience of their safety, tolerability, and suitability for different subgroups of PAH patients, insights that will guide and inform the development of further therapeutics in this disease space.

## AUTHOR CONTRIBUTIONS

PDU wrote the article, BJD WL and NWM reviewed and edited the article, BJD prepared the figures. PDU, WL, and NWM secured funding.
